# A core–shell microneedle system for stable fibroblast delivery in cell-based therapies

**DOI:** 10.1007/s13346-024-01759-8

**Published:** 2024-12-19

**Authors:** Federica Medico, Seungcheol Kim, Sachin S. Surwase, Haoyan Liu, Yeu-Chun Kim

**Affiliations:** 1 Department of Chemical and Biomolecular Engineering, Korea Advanced Institute of Science and Technology (KAIST), Daejeon, 34141 Republic of Korea; 2 Department of Biomedical Engineering, Johns Hopkins University, Baltimore, MD 21287 USA

**Keywords:** Microneedle, Cell delivery, Core-shell structure, GelMA, PLGA, Fibroblasts

## Abstract

Human cells, such as fibroblasts and particularly human mesenchymal stem cells (hMSCs), represent a promising and effective therapeutic tool for a range of cell-based therapies used to treat various diseases. The effective delivery of therapeutic cells remains a challenge due to limitations in targeting, invasiveness, and cell viability. To address these challenges, we developed a microneedle (MN) system for minimally invasive cell delivery with high cellular stability. The MN system comprises a core of gelatin methacryloyl (GelMA) hydrogel embedded with fibroblasts, encased in a polylactic-co-glycolic acid (PLGA) shell that enhances structural integrity for efficient skin penetration. The fabrication process involves UV-crosslinking of the GelMA hydrogel with cells, optimizing both cell encapsulation and structural strength. This MN system achieves over 80% cell viability after seven days in vitro, with the conventional GelMA formulation providing superior stability and cellular outcomes. This platform’s ability to ensure sustained cell viability presents promising implications for future applications in regenerative medicine, wound healing, and localized treatments for skin conditions. This MN system opens new avenues for cell-based therapies, offering a versatile and scalable solution for therapeutic cell delivery.

**Graphical Abstract:**

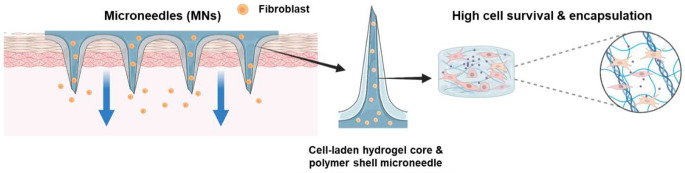

## Introduction

Cell-based therapies are biomedical treatments that involve administering living cells to prevent or treat human disorders, such as cancer, regenerative medicine, and autoimmunity [1]. Some of these therapies have already received FDA-approval and entered the market. For example, cell-based treatments include the delivery of stem cells to treat myocardial infarction and diabetes, and the infusion of chimeric antigen receptor (CAR) T cells to induce regression in advanced lymphoma [2–4].

Currently, cell-based therapies are primarily administered via direct tissue injection or surgical transplant [5]. Although parenteral administration and surgery offer certain advantages, they also have significant drawbacks, such as invasiveness, patient non-compliance, the need for large cell quantities due to low migration efficiency, and the inability to deliver cells precisely to target tissues, which hinders clinical translation and commercialization [6, 7]. Therefore, it is crucial to develop an effective cell delivery system that achieves accurate and precise distribution of viable cells within target tissues.

Microneedles (MNs) are promising platforms for the transdermal delivery of various pharmaceutical compounds [6, 8]. These microdevices consist of arrays of micron-sized needles that can penetrate the stratum corneum (SC) barrier, creating temporary pores to deliver therapeutic molecules into the skin [6, 9, 10]. As minimally invasive devices, they support both local and systemic administration, enhancing patient compliance and allowing for personalized drug delivery with effective therapeutic outcomes [6, 9].

Traditionally, MN arrays have been used to deliver biomolecules such as proteins, peptides, antibodies, vaccines, and nucleic acids [6, 11]. In addition, MNs can deliver nanoparticles and extracellular vesicles that encapsulate bioactive substances through the skin [6]. As a novel approach, MNs have recently been investigated for delivering therapeutic cells. MN-mediated cell delivery has demonstrated therapeutic benefits by enhancing cell penetration and retention, improving tissue targeting accuracy, and reducing systemic toxicity [6]. However, challenges such as prolonged storage, contamination, and maintaining cell viability and functionality remain obstacles to the clinical translation of MN-mediated cell delivery [12, 13].

The hydrogel used for the cell delivery and the MN fabrication requires higher controllability and reproducibility [14]. Gelatin methacryloyl (GelMA) is considered an attractive candidate for the cell loading due to its unique biological and physical properties [15]. GelMA exhibits adjustable rheological and mechanical properties, low cost, good biocompatibility and degradation, low immunogenicity, non-cytotoxicity and chemical versatility [3, 16]. GelMA is produced through a photoinitiated crosslinking reaction between gelatin and methacrylic anhydride (MAA).

GelMA has been predominantly synthesized using the classic or conventional method, as suggested by Van den Bulcke et al. in 2000 [17]. In the conventional method for GelMA synthesis, methacrylate is added dropwise without pH adjustments, resulting in significant variations in GelMA reactivity due to uncontrollable pH conditions. Another synthesis method, known as the one-pot or sequential method, was suggested by Shirahama et al. in 2015. This method sequentially introduces methacrylate into gelatin with pH adjustment to enhance the gelatin reaction [18, 19]. The amount of methacrylate and the pH conditions, which can affect cell viability in the hydrogel, differ between conventional and sequential GelMA. In this research, we synthesized and compared two different types of GelMA hydrogel and analyzed the effectiveness of each GelMA hydrogel MN in cell delivery.

**Fig. 1:**
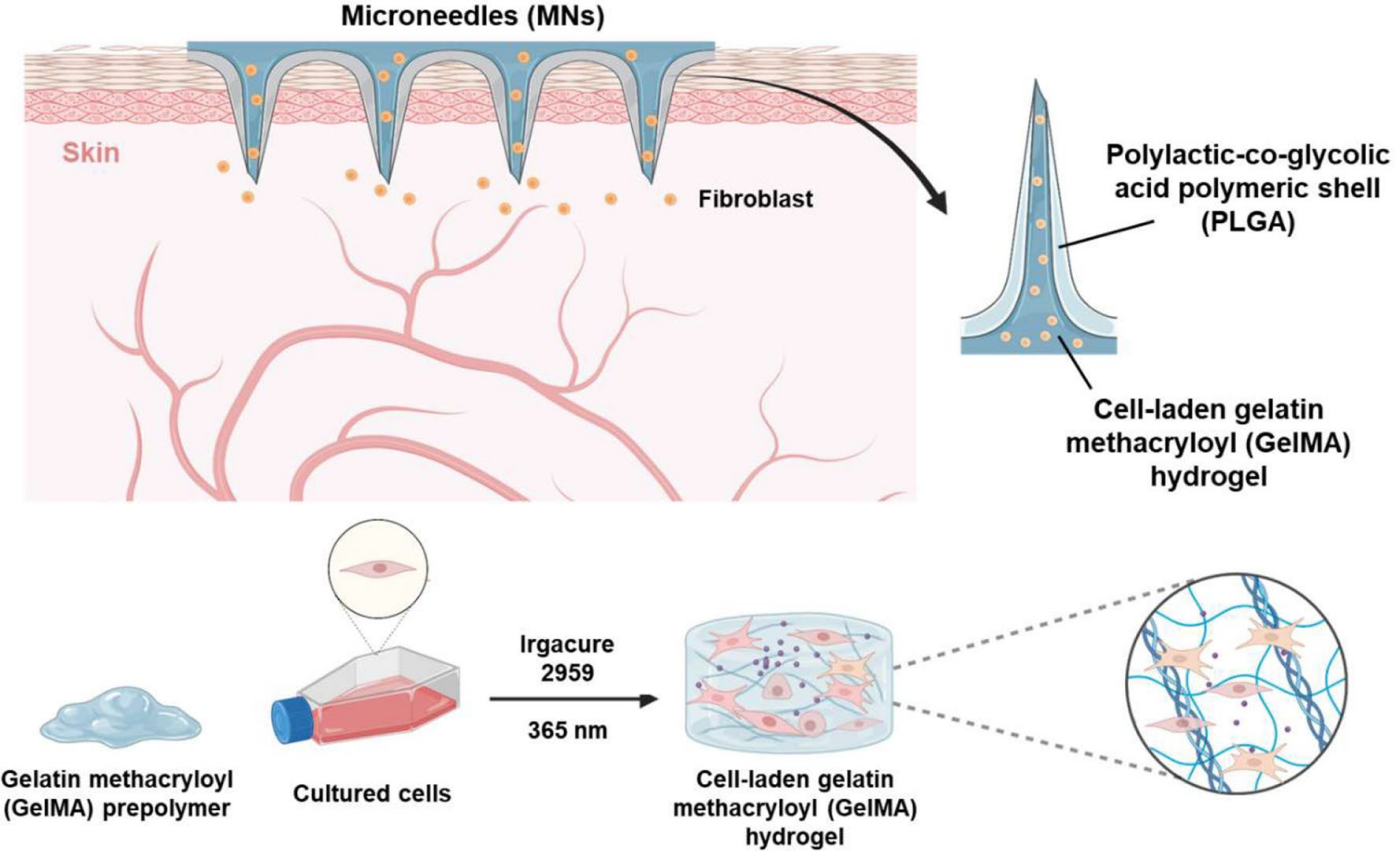
Schematic illustration of the microneedle (MN) system for fibroblast cell delivery, composed of a core of cell-laden-gelatin methacryloyl (GelMA) hydrogel and a polylactic-co-glycolic acid (PLGA) external shell

As shown in Fig. 1, we designed a MN system for fibroblasts delivery, composed of a core of GelMA hydrogel embedded with cells and an external shell made of polylactic-co-glycolic acid 50/50 (PLGA50/50). GelMA is derived from the natural polymer gelatin, which contains cross-linkable methacrylate groups [15]. These groups confer adjustable mechanical and drug release properties to the polymer through controlled crosslinking using ultraviolet or visible light [20]. The presence of moieties in GelMA, such as the tripeptide sequence arginine–glycine–aspartic acid (Arg-Gly-Asp; RGD) and matrix metalloproteinase (MMP) cleavable sites, promotes cell adhesion and inhibits extracellular matrix (ECM) degradation, thereby faithfully replicating the in vivo microenvironment. These properties make GelMA not only an ideal candidate for MN fabrication and other biomedical applications but also one of the best options for cell delivery [21, 22]. Additionally, the rigid PLGA shell facilitates the insertion of the cell-loaded MN into the skin by encapsulating the GelMA-cell mixture [23]. Therefore, this core-shell MN system, with its cell-friendly hydrogel environment and compact coating polymer structure, has the potential to enhance the transdermal delivery efficiency of cells while maintaining high cellular viability.

## Materials and methods

### Cell culture

The NCTC clone 929 and Normal Human Dermal Fibroblasts (NHDF) were cultured in Dulbecco’s modified Eagle’s medium (DMEM) supplemented with 10% fetal bovine serum (FBS) and streptomycin (100 mg/mL). Cells were maintained at 37 °C in humified air with 5% CO_2_ and used for the experiments after 2–3 passages.

### Synthesis of gelatin methacryloyl (GelMA) prepolymer

For the synthesis of GelMA using the conventional method, 10 g type A gelatin powder from acid-cured porcine skin tissue (300 bloom, Sigma-Aldrich) was dissolved in 100 mL of preheated Dulbecco’s Phosphate Buffered Saline (DPBS) (Welgene) at 50 °C for 1 h under constant stirring to obtain a 10% (w/v) gelatin solution. 8 mL of methacrylic anhydride (MAA) (Sigma-Aldrich) was then added dropwise to the gelatin solution and kept under agitation at 50 °C for 2 h. To stop the reaction, warm (40 °C) DPBS was added. To remove residual salts and MAA, dialysis against distilled water was conducted at 40 °C for 7 days using dialysis tubing with a molecular weight cut-off of 10 kDa (Sigma-Aldrich). The solution was filtered using both filter paper and a membrane filter 0.2 μm pore size, frozen to − 80 °C, and lyophilized for at least 3 days [17]. After lyophilization, the obtained gelatin methacryloyl (GelMA), which appeared as a white porous foam, was stored at − 20 °C for further use [24].

In the case of GelMA synthesized using the sequential method, a 10% (w/v) gelation solution was prepared by incorporating 10 g of type A gelation powder into 0.1 M carbonate-bicarbonate (CB) buffer (0.318 g sodium carbonate and 0.586 g sodium bicarbonate in 0.1 L distilled water) at 60 °C. MAA (0.0625 mL) was added every 30 min for a total of 3 h into the solution at 50 °C under constant stirring. The solution was adjusted to pH 9 each time using CB buffer. The reaction was then stopped by reverting the pH to 7.4. After filtration using both filter paper and a membrane filter with a 0.2 μm pore size, the solution was dialyzed for one day at 40 °C using a dialysis bag with 10 kDa molecular weight cut-off. Following three days of lyophilization, the obtained GelMA was stored at − 20 °C for further use.

### Gelation of GelMA hydrogel

The GelMA prepolymer with cell suspension solution was prepared by solubilizing 10% (w/v) dried GelMA and various concentrations of 2-Hydroxy-4′-(2-hydroxyethoxy)-2-methylpropiophenone/Irgacure 2959 (Sigma-Aldrich) in complete growth cell culture media. The mixture was then photocrosslinked for 16 s using a UV curing system (365 nm, CoolWave UV Curing System, Nordson).

### Nuclear magnetic resonance (NMR) spectra of GelMA and gelatin

The substitution of free amino groups on gelatin with methacrylate groups was assessed using ^1^H-NMR analysis conducted with a 600 MHz Liquid NMR Spectrometer (Avance Neo 600, Bruker BioSpin). The samples were prepared by dissolving GelMA and gelatin powder in deuterium oxide (D_2_O) (Sigma-Aldrich) at a concentration of 10 mg/mL at 50 °C.

### Measurement of viscoelastic properties of GelMA

The viscoelastic behavior of hydrogels was investigated using a modular compact rheometer MCR-302 (Anton-Paar), equipped with a 25 mm diameter parallel plate and a gap of approximately 1 mm. Frequency sweep curve measurements were performed at a small strain amplitude (0.1% ) to ensure the measurements remained within the linear viscoelastic region (LVER). Rheological tests were conducted at room temperature (22°C) to observe the storage modulus (G’) and loss modulus (G’’) as functions of a wide range of angular frequencies (ω = 0.1–100 rad/s). GelMA hydrogel discs with a diameter of 35 mm and a thickness of 1 mm were used as samples. Subsequently, at a constant angular frequency (ω = 10 rad/s), the storage modulus (G’) and loss modulus (G’’) were observed as functions of shear strain (γ, % = 0.1–100).

### Fabrication of cell-loaded microneedles

The polydimethylsiloxane (PDMS) mold was prepared by casting the PDMS SYLGARD 184 silicone elastomer kit (elastomer/curing agent = 10:1 by weight), enriched with 1.4–1.5% w/w dimethylsiloxane-(60–70% Ethylene Oxide) block copolymer 20 cSt (Gelest), onto a stainless steel master with an array of 10 × 10 pyramid tips (650 μm in height and 300 μm at the base) [25]. After degassing in a vacuum for 1 h and thermal curing at 70 °C for 24 h, the oppositely replicated PDMS master mold was obtained by carefully peeling it off from the metal substrate.

The PLGA50/50 (Sigma-Aldrich) polymer solution was prepared at a concentration of 40% (w/v) in dimethyl sulfoxide (DMSO). 0.1% (w/v) of sulforhodamine B (RhB) (Sigma-Aldrich) was added to the polymer solution only for the visualization of the PLGA shell. A volume of 50 µL of the polymer solution was cast over the silicone mold and centrifuged at 4000 rpm for 30 min at room temperature (~ 25 °C). The excess layer of the PLGA shell was removed with a blade to create a flat surface, and the mold was placed in a desiccator to allow the shell to solidify. After the initial solidification at room temperature, the casting process was repeated under the same conditions to obtain a uniform layer of the desired thickness. Next, a 50 µL mixture of the GelMA prepolymer and the cell suspension was added to the mold already containing the shell. The mold was then centrifuged at 3500 rpm for 5 min. Following this, the array was crosslinked with 365 nm UV light for 16 s. Finally, commercial double-sided tape was used to peel off the microneedles (MNs).

## Viability and proliferation of cells

LIVE/DEAD™ Viability/Cytotoxicity Kit (Thermo Fisher Scientific), which utilizes calcein-AM and ethidium homodimer-1 (EthD-1), was employed according to the manufacturer’s instructions to assess the cell viability of NCTC clone 929 and NHDF after photoencapsulation within GelMA. Live cells can be distinguished by the presence of intracellular esterase, which converts the nonfluorescent, cell-permeant calcein-AM into the green fluorescent calcein. In contrast, EthD-1 penetrates cells with damaged membranes and binds to DNA, resulting in bright red fluorescence for dead cells. The dye solution was directly added to the photopolymerized GelMA/cells mixture (with a cell density 1 × 10^6^ cells/mL). After incubation for 15 min at 37 °C and three washes with DPBS, the samples were observed using a Zeiss LSM880 confocal laser scanning microscope (excitation for calcein-AM at 494 nm; excitation for EthD-1 at 528 nm) [26].

Quantitative cell viability was measured via CCK-8 assay (Dojindo). After culturing NHDF embedded in a three-dimensional (3D) GelMA structure for the desired duration, the cells were incubated in the dark in serum-free DMEM containing 10% CCK-8 solution at 37 °C for 2 h. As a 2D control, the same number of cells was cultured in the complete growth cell culture media. Following the incubation period, 100µL of supernatant was added to 96-well culture plates, and absorbance was measured by a Gemini XPS microplate reader at 450 nm [27].

## Evaluation of cell encapsulation in the microneedles

NHDF used to assess cell distribution in the microneedles (MNs) via confocal imaging were pre-dyed with Vybrant™ DiD Cell-Labeling Solution (DiIC18(5); 1,1′-dioctadecyl-3,3,3′,3′-tetramethylindodicarbocyanine, 4-chlorobenzenesulfonate salt, Thermo Fisher Scientific). After trypsinization and counting, the cells were suspended in serum-free DMEM at a density of 1 × 10^6^/mL and incubated with 5 µl/mL DiD at 37 °C for approximately 20 min, followed by three washes with DPBS. The labeled cells were then mixed with the GelMA pre-hydrogel, and the MN fabrication was conducted as previously described. Relative images were acquired using a Zeiss LSM880 confocal laser scanning microscope at an excitation wavelength of 646 nm [28].

## Results and discussion

### Characterization of gelatin methacryloyl (GelMA)

During gelation reaction of GelMA, the primary amino and hydroxyl groups on gelatin can be substituted with methacryloyl groups when exposed to UV light in the presence of a photoinitiator (PI). GelMA is responsible for the photochemical crosslinking and the formation of a three-dimensional (3D) network [29]. In the conventional method, a considerably higher ratio of methacrylic anhydride (MAA) to gelatin (0.8 mL/g), with no pH adjustment, and Dulbecco’s Phosphate Buffered Saline (DPBS) as a buffer were employed. In contrast, the sequential method is characterized by the sequential loading of MAA after pH correction every 30 min for 3 h in a carbonate-bicarbonate (CB) buffer system. The ratio of MAA to gelatin is significantly lower, with a maximum of 0.1 mL/g based on the desired degree of substitution, which in our case was set to be 37.5 µL/g.

**Fig. 2:**
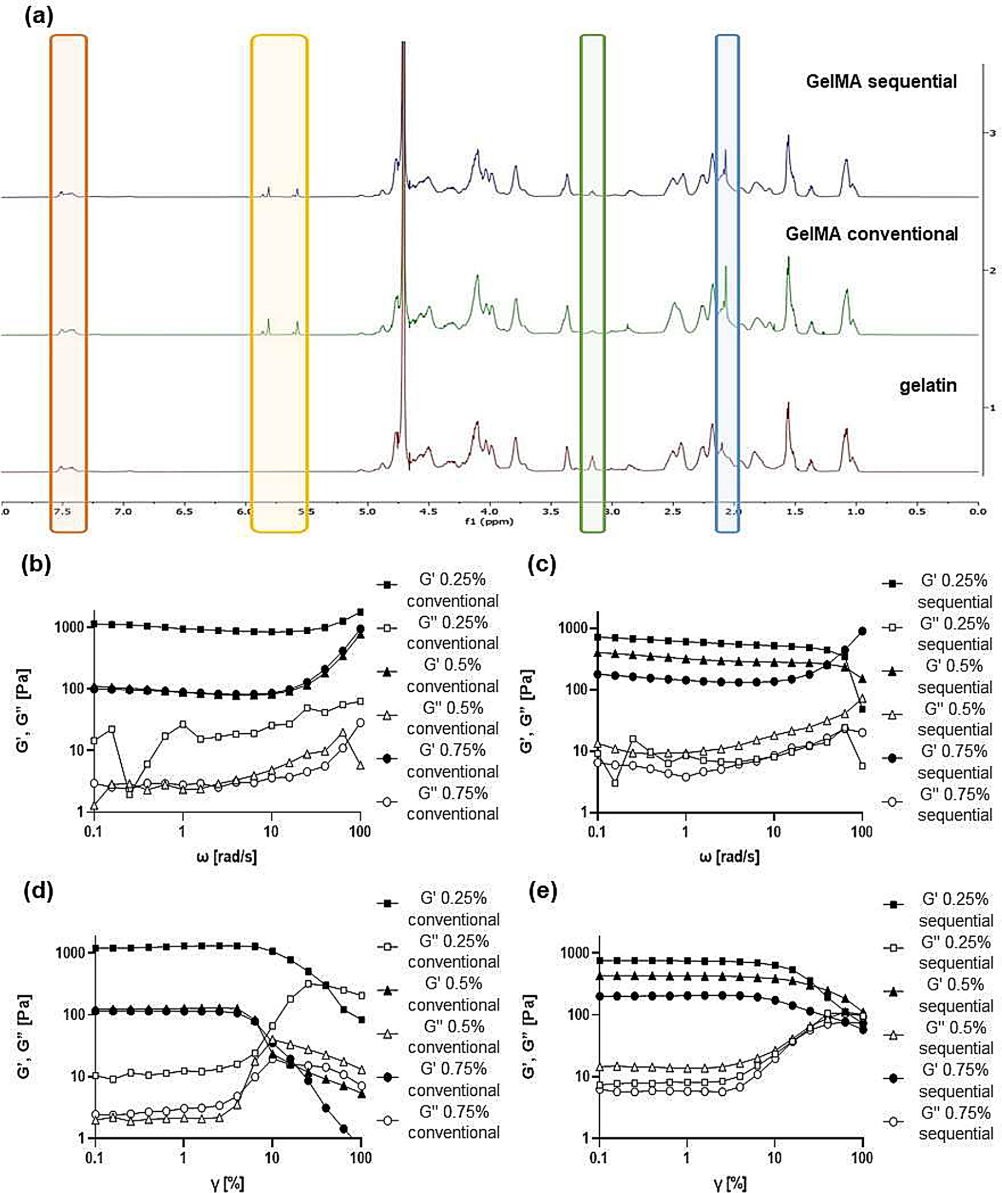
(**a**) ^1^H-NMR spectra, respectively from bottom to top, of unmodified gelatin, gelatin methacryloyl (GelMA) conventional and GelMA sequential. (**b**) Frequency sweep curves related to GelMA conventional and (**c**) GelMA sequential. (**d**) Oscillation strain sweep measurements for GelMA conventional and (**e**) GelMA sequential

The synthesis reaction of the two prehydrogels was confirmed via ^1^H-NMR spectroscopy (Fig. 2a). Compared to the unmodified gelatin spectrum, both GelMA spectra - conventional and sequential - exhibit increased signals at δ = 5.9 and 5.6 ppm and at δ = 2.1ppm, corresponding to the acrylic and methyl protons of the methacrylamide groups of lysine and hydroxylysine, respectively. The intensity of these peaks is directly related to the degree of methacrylation or functionalization (DoF) of the hydrogel. Additionally, a reduced signal is observed at δ = 3.2 ppm, attributed to the methylene protons of unreacted lysine, and this signal is inversely correlated with the DoF [19]. Peaks common to all three spectra (groups unmodified even after the reaction with MAA) were those of phenylalanine’s aromatic rings at δ = 7.5 ppm [30]. Moreover, the DoF of GelMA (considered as the percentage of amino groups from lysine and hydroxylysine in gelatin that have been modified after the methacrylation reaction) was also determined using ^1^H-NMR spectroscopy. The phenylalanine aromatic signal (7.5 ppm), representing the concentration of gelatin, served as the reference integral to normalize all the spectra, while the reduction in signal intensity of the lysine methylene signal in unmodified gelatin and GelMA (3.2 ppm) was analyzed [31].

The DoF of GelMA, both conventional and sequential, was calculated as follows:

$$\begin{aligned}&\text{DoF}[\% ]\cr&\quad =1-\frac{Area(lysine\,methylene\,of\,GelMA)}{Area(lysine\,methylene\,of\,unmodified\,gelatin)}\times 100\end{aligned}$$

The DoF of GelMA conventional was calculated to be 76.9 ± 0.8%, while the DoF of GelMA sequential was found to be 55.4 ± 1.0%. The DoF calculation in the latter case showed results similar to those obtained by Lee et al. using the same MAA/gelatin ratio [18]. For the crosslinking of both types of GelMA in this study, the following conditions were employed: a 10% w/v GelMA suspension, exposure to 365 nm UV light, and Irgacure 2959 as the PI at three different concentrations: 0.25, 0.5 and 0.75% (w/v).

For further characterization of GelMA, we analyzed the elastic (or storage) modulus G’ and the viscous (or loss) modulus G’’ as a function of angular frequency (ω) at 20 ºC. Figure 2b and c show the frequency sweep curves, illustrating the influence of angular frequency (ω; 0.1–100 rad/s) at a constant strain (0.1%) on G’ and G’’ for both conventional and sequential GelMA hydrogels in relation to PI concentration. A small constant strain value was chosen to ensure that measurements were taken within the linear viscoelastic region (LVER). These curves provide a rheological representation of the product’s behavior during its shelf life and use. The dynamic viscoelasticity of a hydrogel in the low angular frequency (ω) range is highly responsive to structural alterations or the development of a network-like arrangement.

The figures reveal a consistent trend across all samples: the storage modulus G’ consistently exceeds the loss modulus G’’ for each PI concentration. This suggests that all hydrogels behave as viscoelastic solids, exhibiting predominant elastic behavior over viscous behavior due to internal links (chemical bonds and/or physical-chemical interactions). There is negligible alteration in the values of G’ and G’’ across most of the different angular frequencies applied, exhibiting behavior resembling a rubbery plateau. These findings suggest effective crosslinking of the hydrogels, with angular frequency having minimal impact on altering their predominantly elastic behavior. This attribute is important for the practical applications of hydrogels. Additionally, for both types of GelMA hydrogels, 0.25% (w/v) PI concentration shows higher elasticity than 0.5 and 0.75% (w/v), which are quite similar to each other. This may lead to more crosslinked networks within the hydrogel, indicating stronger solid-like properties and greater mechanical rigidity [32, 33].

Figure 2c and d illustrate oscillation strain sweep measurements, depicting how G’ and G” vary in a strain range of 0.1–100% at a constant angular frequency of 10 rad/s. These trends are consistent across all tested hydrogels. Within a certain range of deformation, both moduli remain constant with increasing shear strain amplitude, with G’ exceeding G”. This phase defines the LVER, associated with elastic behavior without microstructural changes in the hydrogel, lasting until a critical shear strain amplitude is reached. Beyond this point, there is a noticeable decrease in G’, linked to the polymer’s yield strain, which marks the onset of irreversible microstructural alterations, while the G’’ modulus constantly increases. At higher deformations, a critical strain, known as the flow point, is reached where G’ equals G’’, indicating that the hydrogel transitions to a liquid-mimicking behavior, dissipating significant energy and leading to complete microstructure destruction [34, 35].

Both GelMA conventional 0.5% and 0.75% (w/v) show a flow point at a strain around or slightly above 10%, while in the case of GelMA conventional 0.25% (w/v), it is closer to 100%. For all the GelMA sequential hydrogels, the flow point is observed at a strain close to 100%. With further increases in strain, the viscous modulus (G′′) becomes dominant over the elastic modulus (G′) for all the hydrogels.

### Characterization of GelMA-cell mixture for cell viability

To prevent cell damage and ensure sufficient cell viability, selecting optimal photocrosslinking conditions is essential: the wavelength of emitted light must match the PI’s absorption spectrum, while the PI concentration and crosslinking time must be carefully optimized. Studies have shown that cell viability is inversely proportional to the concentration of PI and UV exposure time [36, 37]. In this study, we used UV light at 365 nm with a polymerization time of 16 s for all the experiments. There is substantial evidence indicating that increased UV exposure negatively impacts cell migration, intercellular interactions, and nutrient diffusion due to increased stiffness and reduced pore size, especially in highly functionalized GelMA hydrogels. Therefore, we empirically selected 16 s as the minimum UV exposure time required to effectively polymerize all GelMA hydrogels [35, 38].

**Fig. 3:**
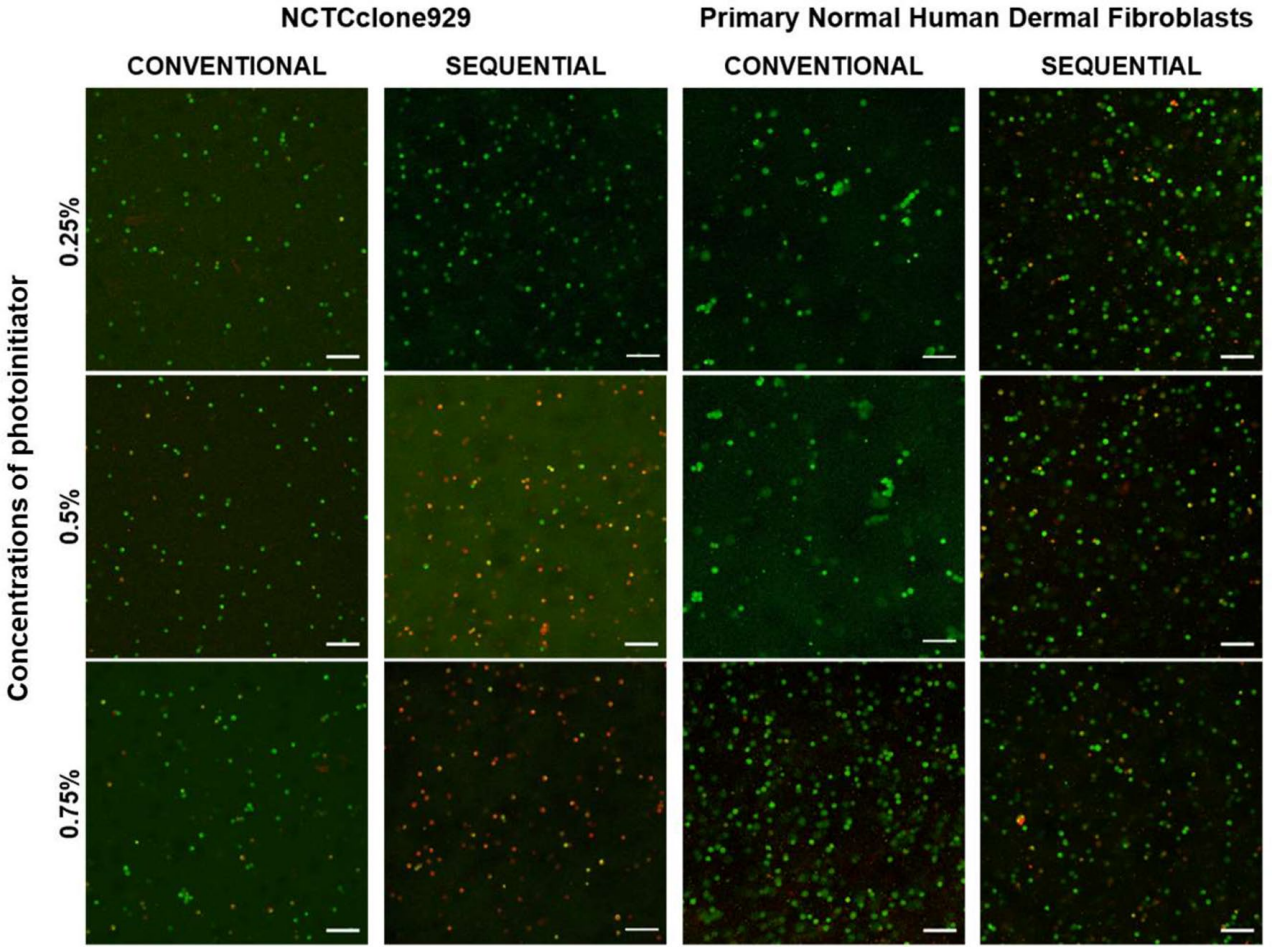
In vitro live/dead assay results using a confocal microscope. The two columns on the left are related to NCTC clone 929, and the two columns on the right refer to Primary Normal Human Dermal Fibroblast (NHDF). Each cell line was evaluated from the gelatin methacryloyl (GelMA) made from conventional and sequential method. In addition, the cellular viability of each cell line was investigated in hydrogels with different concentrations of photoinitiator (PI) (scale bars = 100 μm)

Assessing cell viability is a crucial factor in evaluating the functionality of the synthesized hydrogels and their suitability for cell delivery applications. First, cell viability was assessed qualitatively using a live/dead assay to compare conventional and sequential GelMA hydrogels with PI concentrations of 0.25, 0.5, and 0.75% (w/v) (Fig. 3). The experiment was conducted with two independent cell lines - murine (NCTC clone 929 or L-929) and human fibroblasts (NHDF) - encapsulated in 10% GelMA. Cells were exposed to a solution containing calcein-AM and ethidium homodimer-1 (EthD-1) for 15 min at 37 °C. Live cells were indicated by green fluorescent calcein, while dead cells were indicated by red fluorescent EthD-1. In the GelMA conventional groups, the majority of both murine and human fibroblasts appeared viable, whereas in the GelMA sequential groups, especially in the NCTC clone 929 samples, a higher number of dead cells was observed. With higher PI concentration in the GelMA sequential hydrogel, the viability of NCTC clone 929 cells decreases, indicating that the PI concentration should be carefully optimized for cell-laden hydrogel in the MN system.

**Fig. 4:**
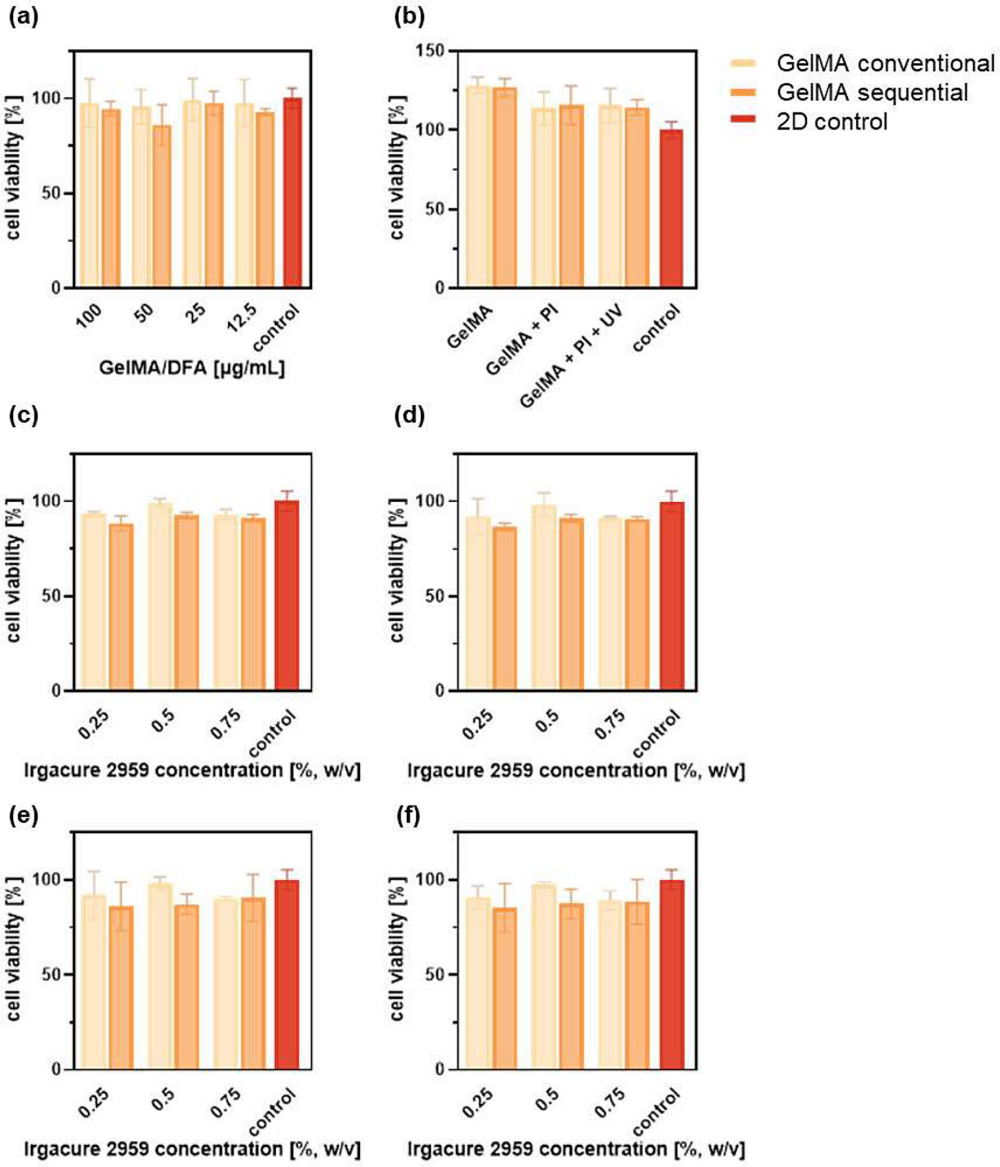
Results of cell counting kit-8 (CCK-8) assay on Normal Human Dermal Fibroblast (NHDF). (**a**) Biocompatibility study of gelatin methacryloyl (GelMA) using the serial dilution method at 24 hours. (**b**) Evaluation of the NHDF cell viability after being exposed to the various fabrication steps involved in the MN assembly for 24 hours. (**c**-**f**) Evaluation of the NHDF cell viability after GelMA fabrication on day (**c**) 1, (**d**) 3, (**e**) 5 and (**f**) 7

Subsequently, the cell counting kit-8 (CCK-8) assay was utilized to evaluate fibroblast cell viability and proliferation within the GelMA hydrogels. To assess biocompatibility, NHDF cells were embedded in matrices containing various concentrations of GelMA hydrogel, obtained through serial dilution. After a 24-hour exposure to complete growth cell culture medium (DFA) mixed with methacrylated hydrogel in decreasing GelMA/DFA ratios from 100 to 12.5 µg/mL, a viability test was performed (Fig. 4a). The CCK-8 assay results indicated similar cell survival rates between the medium + hydrogel mix groups and the medium-only (2D control group), with no significant differences observed across different GelMA concentrations. These results confirm that, after 24 h, the hydrogels are non-toxic to NHDF compared to the untreated control, suggesting that all GelMA hydrogel concentrations are fully biocompatible and do not impede cell proliferation. For further experiments, the highest concentration tested, 100 µg/mL (corresponding to 10% GelMA hydrogel), was used due to its mechanical suitability and its proven cell-confluence compatibility, as previously reported in the literature [39].

As noted, many conventional microneedle (MN) fabrication methods are too harsh for cell delivery, often impairing cell viability and functionality. To ensure that none of the components used in our MN fabrication process were cytotoxic, the CCK-8 assay was also conducted on NHDF cells exposed to each condition applied progressively during MN fabrication (GelMA only, GelMA + 0.5% (w/v) photoinitiator without and with UV exposure). After 24 h, survival rates remained higher than those of the untreated control group (2D culture in medium only), indicating non-toxicity for human fibroblasts (Fig. 4b).

The CCK-8 assay was then used to evaluate and compare cell proliferation in 3D-cultured NHDF cells (embedded in a GelMA matrix) and 2D-cultured NHDF after GelMA fabrication on day 1, 3, 5, and 7 (Fig. 4c-f), which is critical for determining the GelMA candidate with optimal performance. All groups maintained viability above 80% after 7 days. By analyzing and combining these results with the live/dead assay data, we identified GelMA prepared by the conventional method with 0.5% photoinitiator as the most effective candidate, demonstrating the highest cell viability and selected for subsequent experiments.

These findings highlight the importance of factors influencing GelMA’s physical and mechanical properties, including PI concentration and DoF. Higher DoF typically increases hydrogel stiffness and decreases pore size [40], which can limit cell mobility and restrict oxygen and nutrient diffusion. In our study, GelMA produced by the conventional method achieved an optimal DoF, providing the mechanical properties needed to support MN fabrication while ensuring cell survival, as confirmed across all viability assays.

### Fabrication of core-shell MN array

**Fig. 5:**
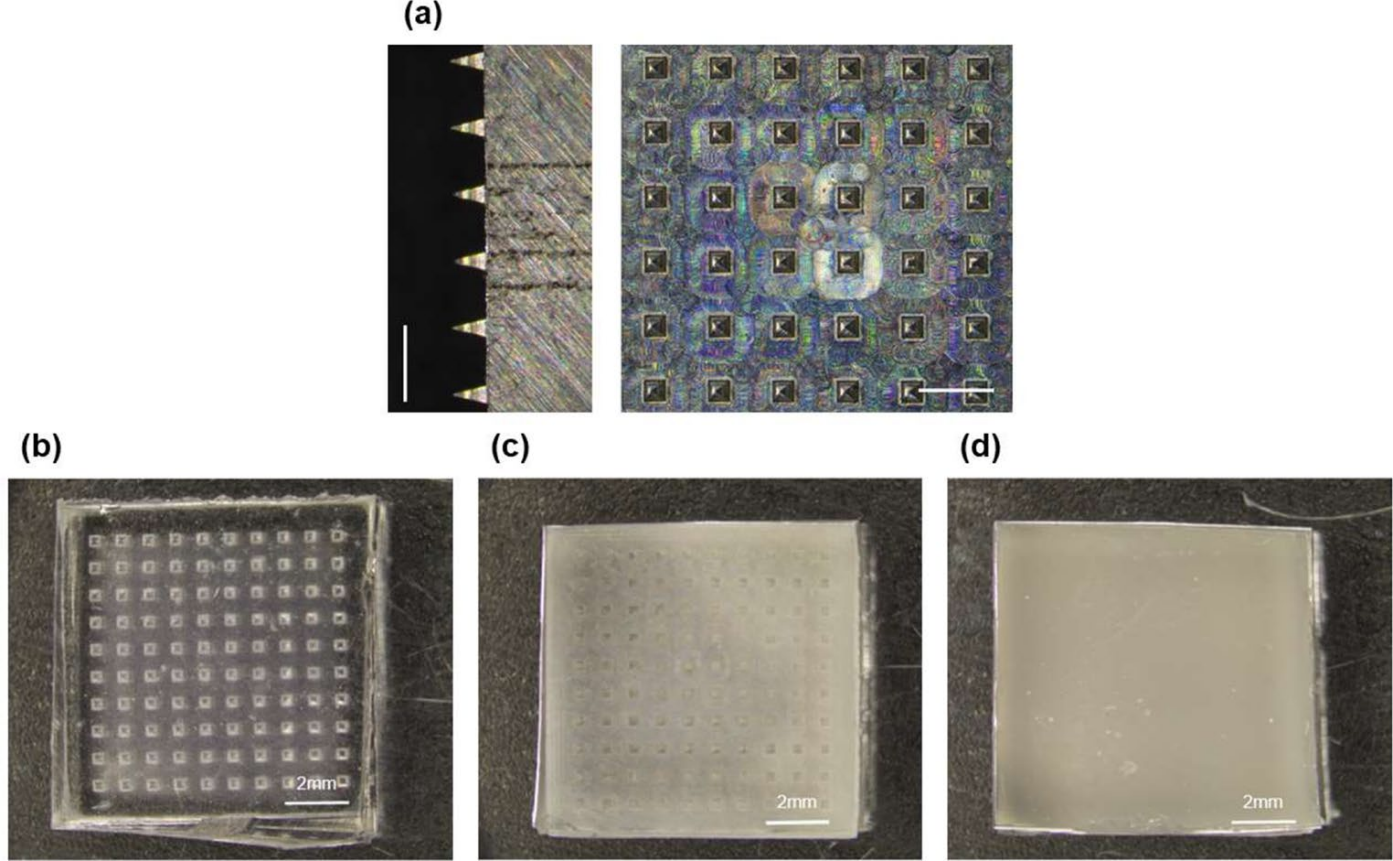
(**a**) Stereo microscopy images (side and top view) of the positive steel master mold (scale bars = 1 mm). (**b**) Stereo microscopy image of the traditional negative silicone (PDMS) mold, (**c**) the front part of the new negative silicone (PDMS + PDMS-PEG block copolymer) mold, and (**d**) the back (scale bars = 2 mm)

To create a positive mold for the MN array, we used a pyramid-shaped steel mold with a 10 × 10 array configuration. Each tip on the mold measured 300 μm in width, 300 μm in length, and 650 μm in height, with a center-to-center spacing of 960 μm (Fig. 5a).

The negative mold was then prepared via micromolding, combining a polydimethylsiloxane (PDMS) base with a curing agent in a 10:1 weight ratio (Fig. 5b). However, the high hydrophobicity of traditional PDMS molds posed challenges in hydrogel penetration into the microcavities, preventing successful needle formation. To enhance wettability and optimize the contact angle between the hydrogel and the PDMS mold, we adopted a technique introduced by Gökaltun et al. (2019), which involves adding a smart copolymer composed of poly(ethylene glycol) (PEG) and PDMS segments. When blended with PDMS, this copolymer enhances the hydrophilicity of the mold and demonstrates biocompatibility, with the modification remaining effective for at least twenty months [25]. In our project, we added PDMS-PEG block copolymer (BCP) to the PDMS prepolymer before curing, at a concentration of 1.5% (w/w), which we empirically determined as optimal for negative mold fabrication (Fig. 5c and d).

**Fig. 6:**
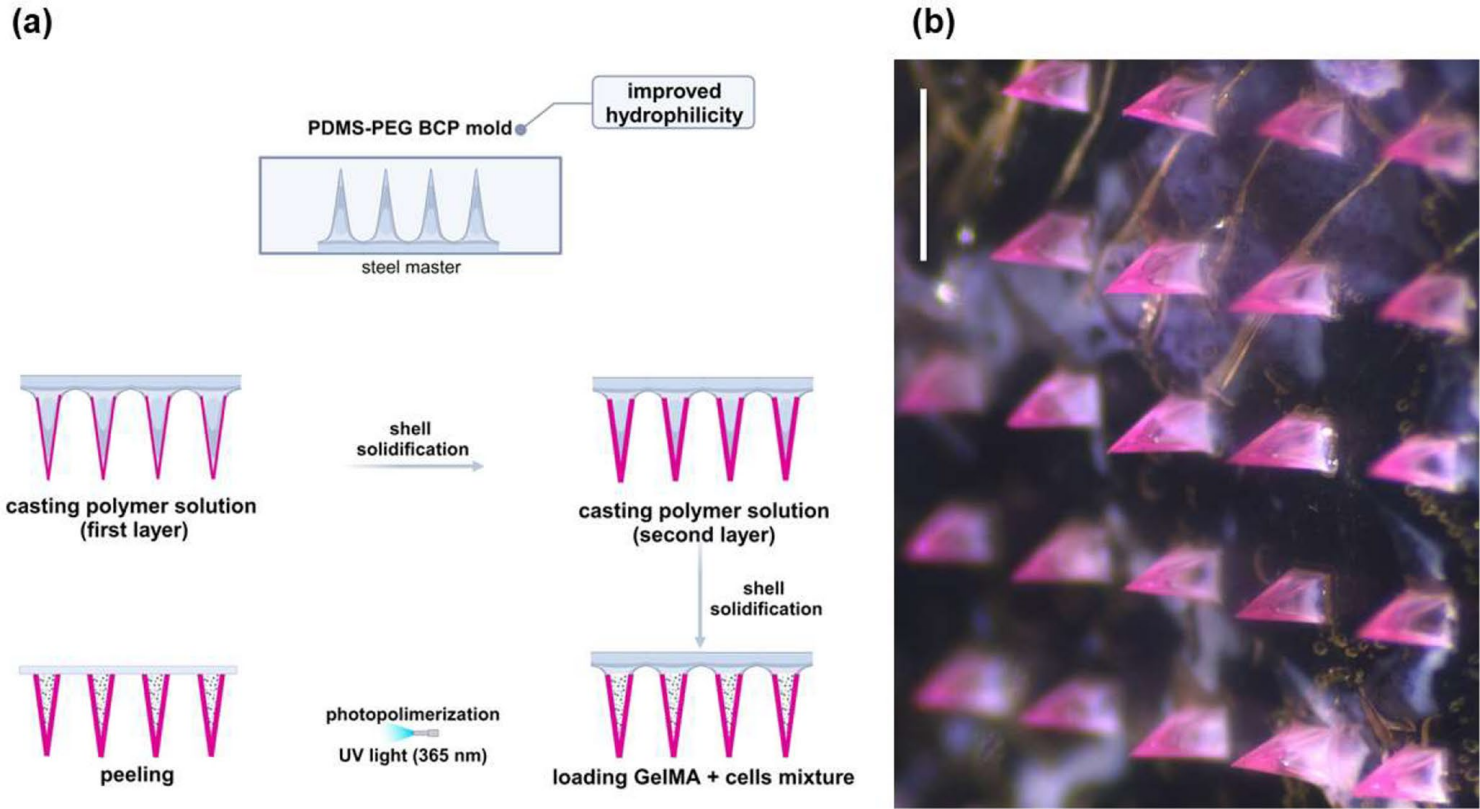
(**a**) Overall fabrication process of the core-shell microneedle (MN) structure. (**b**) Stereo microscopy image of MN array loaded with cell-embedded gelatin methacryloyl (GelMA) inside the Rhodamine B-dyed poly(D, L-lactide-co-glycolide) (PLGA) shell (scale bars = 1 mm)

In Fig. 6a, the overall fabrication process of the core-shell MN structure is illustrated. A 40% (w/v) solution of poly(D, L-lactide-co-glycolide) (PLGA) 50:50 was prepared in dimethyl sulfoxide (DMSO) to form the shell polymer solution. This solution was pipetted onto the modified silicone mold, followed by centrifugation. After the solvent evaporated, the casting process was repeated to achieve a shell of uniform thickness. Next, the GelMA-NHDF mixture was centrifuged to encapsulate the cells within the PLGA shell, and, after removing any excess material, photocrosslinking was applied. To facilitate removal of the MN array, double-sided tape was used to extract the final MN from the mold (Fig. 6b). Rhodamine B (RhB), a dyeing agent, was added to the PLGA solution to visualize the formation of the PLGA shell within the MN.

**Fig. 7:**
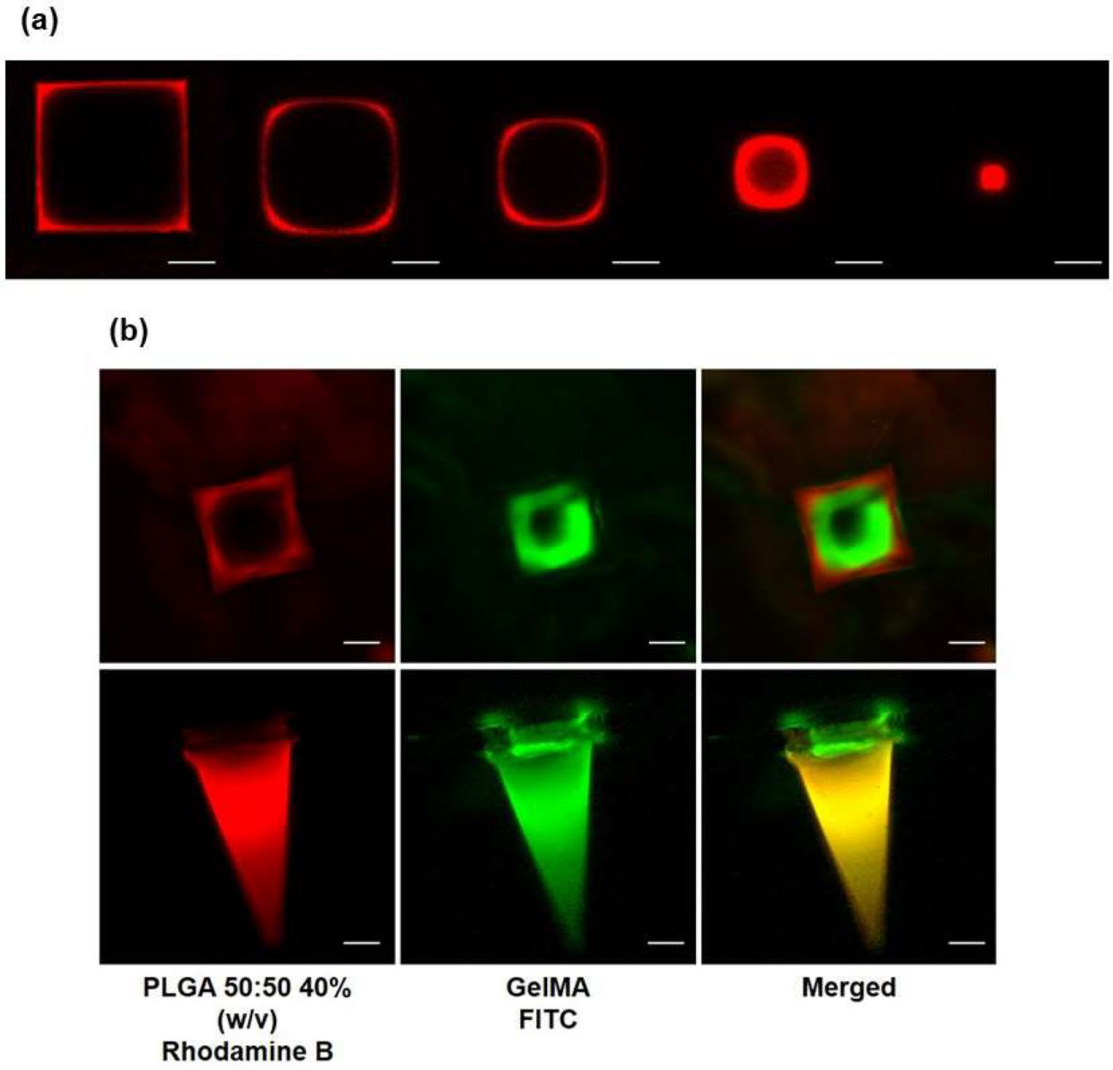
Confocal microscopic images related to the core-shell microneedle (MN) array. (**a**) Cross sections along the length of the rhodamine B (RhB)-dyed poly (D, L-lactide-co-glycolide) (PLGA) shell. (**b**) Base view and side view of of RhB-dyed PLGA (red) and FITC-labeled gelatin methacryloyl (GelMA) (green) resulted to be both evenly spread along the whole length of the MN (scale bars = 100 μm)

As shown in Fig. 7a, cross-sectional images along the needle length reveal the uniform formation of the red PLGA shell, with an even diameter maintained from top to bottom. To confirm the successful incorporation of hydrogel within the needles, thus constituting the core, GelMA was covalently conjugated with fluorescein (FITC) and characterized using fluorescence confocal microscopy (wavelength: 500 ~ 650 nm). As illustrated in Fig. 7b, the RhB-dyed PLGA (red) and FITC-labeled GelMA (green) were evenly distributed throughout the length of the MN, verifying consistent morphology and the sharpness of the needle tips.

**Fig. 8:**
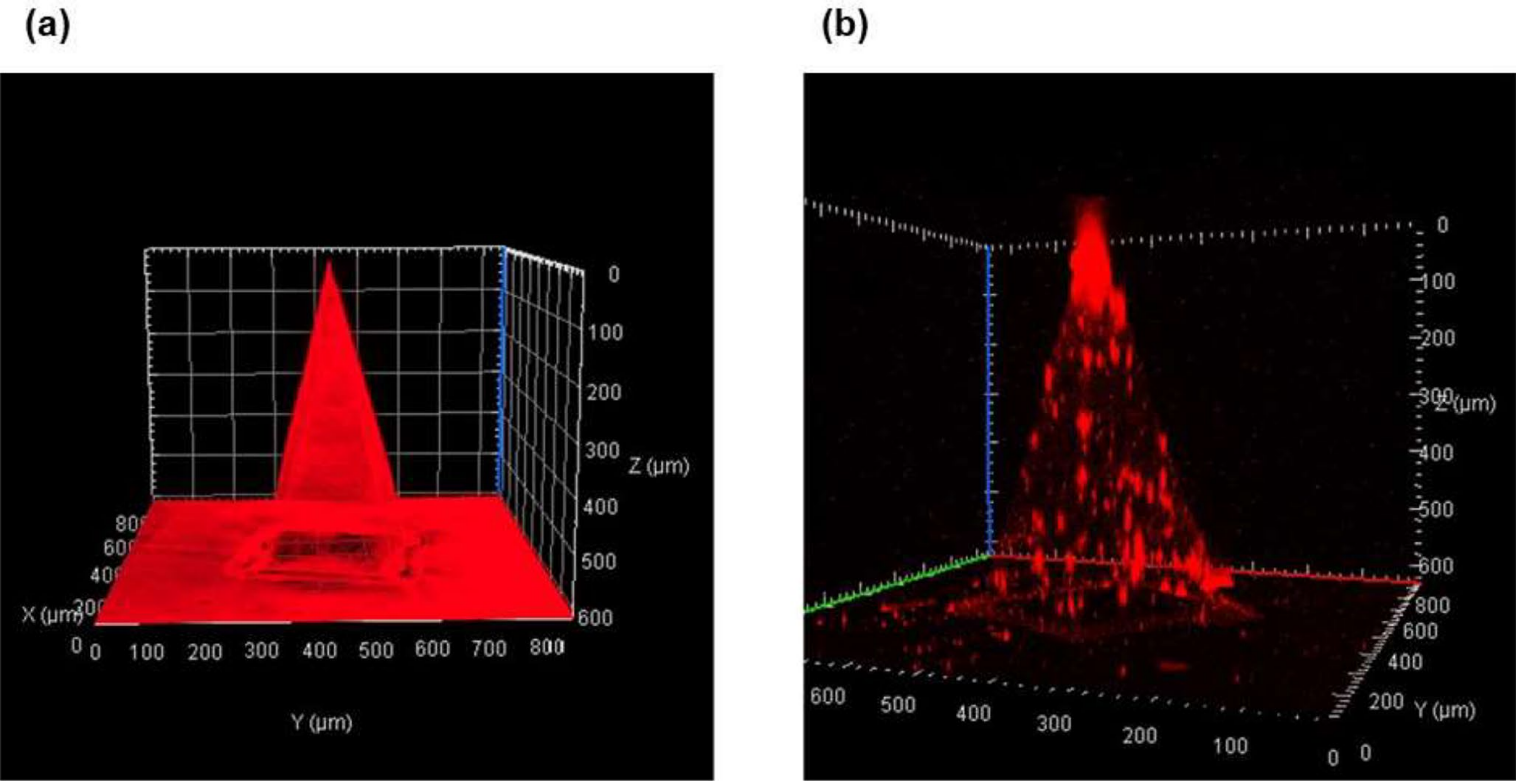
Three-dimensional fluorescence images related to the core-shell microneedle (MN) array. (**a**) Side view of rhodamine B (RhB)-dyed poly(D, L-lactide-co-glycolide) (PLGA) shell. (**b**) Side view of DiD-labeled Normal Human Dermal Fibroblasts (NHDFs) loaded in the MN

The square-based pyramid shape with a sharp tip, characteristic of the RhB-dyed PLGA shell, was also confirmed through 3D imaging (Fig. 8a). To further verify successful cell loading and determine cell distribution within the MN shell, DiD-labeled NHDF-laden MNs were prepared. Red fluorescent signals, representing the morphology and positioning of the labeled cells, were observed within the MNs (Fig. 8b). DiD was chosen to visualize cells non-invasively, avoiding overlap with the autofluorescence of the PLGA shell. A uniform distribution of red signals throughout the MNs indicated that cells were randomly dispersed within the MN matrix, with no clustering or disintegration observed. These images collectively confirm the successful fabrication of the MNs.

## Conclusion

In this study, we developed an innovative core-shell microneedle (MN) array for fibroblast delivery. The poly(D, L-lactide-co-glycolide) (PLGA) shell provides structural rigidity, while the extracellular matrix (ECM)-mimicking gelatin methacryloyl (GelMA) hydrogel, which is biocompatible and biodegradable, supports cell survival despite the challenges encountered during fabrication. Across all groups, cell viability remained above 80% even after seven days, with GelMA prepared by the conventional method using 0.5% photoinitiator demonstrating the highest viability. In addition, we successfully fabricated the core hydrogel-polymer shell structure MNs with a consistent morphology and uniform cell distribution [41].

As previously mentioned, many researchers have shown the advantages and potential of MN-mediated cell delivery. This MN system offers a less invasive, more controlled approach to administering cell-based therapies through the skin, garnering positive responses from both patients and specialists. Cell-based therapies using various cell types can effectively restore or repair damaged tissues, addressing a broad range of diseases and conditions. This system has potential applications beyond fibroblast delivery, including treatment of vitiligo, scar and burn remodeling, wound and skin ulcer repair, immunotherapy, cancer vaccines, and hormone modulation [6, 7, 42, 43].

## Data Availability

The datasets generated and/or analyzed during the current study are available from the corresponding authors on reasonable request.

## Declaration

provided our consent for the publication.
